# Association of Alcohol Intake and Semen Parameters in Men With Primary and Secondary Infertility: A Cross-Sectional Study

**DOI:** 10.3389/fphys.2020.566625

**Published:** 2020-09-11

**Authors:** Shun Bai, Yangyang Wan, Lu Zong, Wei Li, Xiangdong Xu, Yun Zhao, Xuechun Hu, Yanzhen Zuo, Bo Xu, Xianhong Tong, Tonghang Guo

**Affiliations:** ^1^Reproductive and Genetic Hospital, The First Affiliated Hospital of USTC, Division of Life Sciences and Medicine, University of Science and Technology of China, Hefei, China; ^2^Department of Urology, The First Affiliated Hospital of USTC, Division of Life Sciences and Medicine, University of Science and Technology of China, Hefei, China

**Keywords:** alcohol consumption, primary infertility, secondary infertility, semen quality, sperm concentration

## Abstract

Alcohol consumption has commonly been associated with semen parameters. However, the association between alcohol intake and semen parameters in primary and secondary infertile men remains unclear. In this study, 776 infertile men from China were grouped according to alcohol intake: abstainers, moderate drinkers (<9 units/week, up to approximately 100 g of ethanol) and heavy drinkers (≥9 units/week). Semen parameters, including semen volume, sperm concentration, total sperm count, progressive motility and normal morphology were investigated. Alcohol consumption and other lifestyle factors were assessed by questionnaire. Logistic regression models were applied. There was no significant association between alcohol consumption and semen parameters in men with primary infertility. Smaller testis volumes and lower sperm concentrations were found among moderate and heavy drinkers in the secondary infertility group than among abstainers. After adjustment for potential confounders, men with secondary infertility and heavy alcohol consumption had a higher risk of abnormal sperm concentrations (OR = 3.72; 95% CI, 1.04, 13.37). These findings suggest that alcohol intake may decrease sperm concentrations in men with secondary infertility, whereas no association was found in men with primary infertility. It may be beneficial for clinicians to advise male patients with secondary infertility who are seeking fertility treatment to avoid heavy alcohol consumption.

## Introduction

A reduction in sperm concentration of approximately one-half was reported in a meta-analysis of Asian men over the past 35 years, and low semen quality was associated with male subfertility (Sengupta et al., 2017). According to information collected by Boivin et al. (2007) approximately 15% of couples of reproductive age suffer infertility. In Northeast China, 18% male-only factors and 20% combined male and female infertility were found in assisted reproductive technology (ART) patients (Fang et al., 2018). Infertility patterns are conventionally divided into primary and secondary types; the former indicates a failure to become pregnant after 1 year, and the latter indicates an inability to become pregnant after 1 year after previously having fathered one or more biological children (Mohamed et al., 2017). Previous studies have shown that the proportion of primary infertility decreased while the proportion of secondary infertility increased in China from 2012 to 2016 (Fang et al., 2018).

The etiology of male infertility involves both intrinsic (such as genetic and congenital disorders) and extrinsic factors (such as lifestyle and environmental factors) (Wang et al., 2018). In recent years, both patients and infertility specialists have increased the attention focused on male infertility through modifiable lifestyle behaviors. Several articles have reported that semen quality in men with primary or secondary infertility could be influenced by lifestyle factors, including dietary patterns, physical activity levels, occupational characteristics, smoking, and alcohol consumption (Jensen et al., 2014; Boeri et al., 2019). The negative impacts of an unhealthy lifestyle may induce increasingly greater loss of semen quality in men with secondary infertility, meaning that poor lifestyle habits are more commonly associated with secondary than primary infertility (Katib et al., 2014). Accordingly, intrinsic factors are less likely to play a role in secondary infertility (because they have proven to be fertile previously) and thus extrinsic factors may play a larger role. However, to the best of our knowledge, no study has yet investigated the potential differences in alcohol consumption between men with primary and men with secondary infertility. It is therefore important to analyze the impact of different lifestyles on semen quality in men with primary and men with secondary infertility.

Studies have focused on analyzing the effect of alcohol intake on spermatogenesis and male fertility, as drinking is very common in adult men (Ricci et al., 2018). Several authors have found a negative association between drinking and semen parameters (such as semen volume, total sperm count, progressive motility, and normal morphology) (Boeri et al., 2019). However, other studies have provided inconsistent results. For example, a positive correlation was confirmed between moderate alcohol consumption (4–7 units/week) and sperm quality (Ricci et al., 2018).

Due to differences in drinking habits across study populations, the current evidence related to the impacts of alcohol on individual sperm parameters remains controversial. The purpose of this study was to investigate the relationship between alcohol intake and semen quality in men with primary or secondary infertility in China according to the 5th World Health Organization (WHO) guidelines (WHO, 2010).

## Participants and Methods

### Patients

**FIGURE 1:**
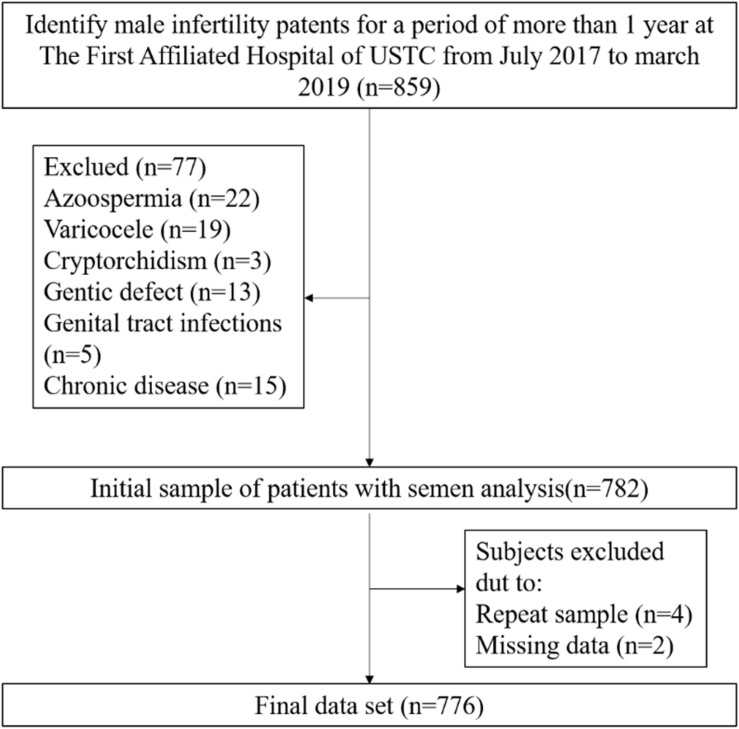
Flow diagram for the selection of the eligible study population.

In the present study, data from men with primary or secondary infertility were collected between July 2017 and March 2019 from a single center of reproductive medicine in Anhui, China. After eliminating the infertility caused by female factors and including only women younger than 40 years of age with normal menstrual cycles, ovulation, and uterine cavity, infertile patients were included in this study if they had solely male infertility. This was a cross-sectional study conducted to assess the association of alcohol consumption and semen quality in men with primary and secondary infertility (Figure 1). Initially, the study population consisted of 859 men. During data verification, a population of 77 men were excluded due to azoospermia (*n* = 22), varicocele (*n* = 19), cryptorchidism (*n* = 3), genital tract infections (*n* = 5), chronic severe debilitating medical illnesses (*n* = 15) or genetic defects related to the male reproductive tract (*n* = 13). Men who were repeat participants (*n* = 4) or who did not provide complete information about alcohol consumption (*n* = 2) were excluded. Ultimately, 776 men were enrolled in this study and underwent more than one semen analysis, both showing abnormal semen parameters according to the 5th WHO criteria. This study was approved by The First Affiliated Hospital of USTC Ethical Committee, Anhui, China.

### Semen Parameters

Semen volume (mL) was assessed by weighing after an abstinence period of 2–7 days. Semen samples were liquefied at 37°C for 30 min and analyzed in accordance with the WHO guidelines (WHO, 2010). For semen analysis, semen volume (mL) was measured via collection tube. Sperm concentration and progressive motility were measured by computer-assisted sperm analysis (CASA) (Weili, Beijing, China). Sperm morphology was determined through Diff-Quick staining (Anke Biotechnology, Hefei, China) and the assessment of approximately 200 spermatozoa per sample. Antisperm antibody (AsA) measurements were performed with the mixed antiglobulin reaction (MAR) method (Anke Biotechnology, Hefei, China). According to WHO recommendations, round cells were recognized as leukocytes by the peroxidase test using benzidine (Anke Biotechnology, Hefei, China).

### Clinical Characteristics

The baseline assessment included a detailed physical examination. Body mass index (BMI) was calculated for every patient according to WHO BMI cut-offs in China (WHO Expert Consultation, 2004). Follicle-stimulating hormone (FSH), luteinizing hormone (LH), total testosterone (TT), 17-β-estradiol (E2), and prolactin (PRL) levels were measured by chemiluminescence microparticle immune assays. Testicular volume was measured by testicular ultrasonography. The testicular volume was calculated using the Lambert formula: length × width × height × 0.71. Spermatic vein reflux was detected by color-duplex ultrasound.

### Alcohol Intake Data Collection

A questionnaire that included alcohol consumption was completed by each of the patients. All data on alcohol consumption were obtained as weekly intake. The patients were grouped as follows: abstainers = non-alcohol consumers; moderate drinkers = up to 1 L of wine or 2.64 L beer per week (8 units, approximately 100 g of ethanol); and heavy drinkers = more than 1 L of wine or 2.64 L beer per week.

### Statistical Analyses

Qualitative variables were described as frequency and percentage, and quantitative variables were described as mean ± standard deviation (SD) if normally distributed and medians [interquartile range (IQR)] if not. To compare variables between two groups, Pearson’s chi-square test and Student’s *t*-test were used for the qualitative and quantitative variables with normal distribution, respectively; the Mann–Whitney *U* test was utilized for parameters with non-normal distribution. For comparisons of three groups, chi-square tests and ANOVA were employed for the qualitative and quantitative variables with normal distribution, respectively, while the Kruskal–Wallis test was used for non-normal distribution. Abnormal semen parameters according to the WHO (2010) recommended standards were listed as follows: semen volume < 1.5 mL, sperm concentration < 15 × 10^6^/mL, progressive motility < 32 × 10^6^/mL and motility morphology < 4%. Logistic regression was used to analyze the association between alcohol consumption and semen parameters. The reference group comprised the study participants who were abstainers (OR = 1). This study presented two analyses: an unadjusted model and an adjusted model that included age, BMI, abstinence time, smoking, duration of infertility, nighttime snack intake, dietary habits, and sleep time. Statistical analyses of all data were considered significant at *P* value of less than 0.05. All statistical analyses were performed using SPSS version 17 (SPSS Inc., Chicago, IL, United States).

## Results

The characteristics of the participants with primary or secondary infertility are shown in Table 1. The mean age of the men with primary infertile or secondary infertility included in this study was 31.4 ± 5.4 years. The BMI, testis volume, leukocytes and AsA were not significantly different between the primary and secondary infertility groups. The men in the secondary infertility group were significantly older than the men in the primary infertility group and serum TT levels were lower among men with secondary infertility. Significant differences were found in the duration of infertility of the cases; compared with the secondary infertility group, the primary infertile group endured longer periods of infertility. Interestingly, the frequency of nighttime snack intake and sleep time in the primary infertile group was significantly higher than that in the secondary infertility group. In addition, lower alcohol intake, sperm concentration and sperm progressive motility were observed in the men with primary infertility when compared with the men with secondary infertility.

**TABLE 1:** Characteristics and descriptive statistics of the whole cohort.

**Clinical characteristics**	**Total (*n* = 776)**	**Primary infertile men (*n* = 544)**	**Secondary infertile men (*n* = 232)**	***P***
Age (year), mean ± s.d.	31.4 ± 5.4	29.9 ± 4.5	35.1 ± 5.5	<0.001
<40, n (%)	699 (90.1)	522 (96.0)	177 (76.3)	<0.001
≥40, n (%)	77 (9.9)	22 (4.0)	55 (23.7)	
BMI (kg/m^2^), mean ± s.d.	20.9 ± 2.8	20.8 ± 2.9	21.0 ± 2.6	0.30
<24, n (%)	667 (86.0)	464 (85.3)	203 (87.5)	0.42
≥24, n (%)	109 (14.0)	80 (14.7)	29 (12.5)	
Left testis volume (cm^3^), mean ± s.d.	13.9 ± 4.3	13.8 ± 4.2	14.4 ± 4.5	0.36
Right testis volume (cm^3^), mean ± s.d.	15.0 ± 4.7	14.8 ± 4.7	15.4 ± 4.8	0.46
**Nation, n (%)**				
Han	768 (99.0)	538 (98.9)	230 (99.1)	0.75
Other	8 (1.0)	6 (1.1)	2 (0.9)	
**Education, n (%)**				
Primary school	16 (2.1)	10 (1.8)	6 (2.6)	0.25
Junior high school	162 (20.9)	109 (20.0)	53 (22.8)	
High school	153 (19.7)	101 (18.6)	52 (22.4)	
College/University	445 (57.3)	324 (59.6)	121 (52.2)	
**Duration of infertility, n (%)**				
1 year	465 (59.9)	291 (53.5)	174 (75.0)	<0.001
2 years	168 (21.7)	142 (26.1)	26 (11.2)	
≥3 years	143 (18.4)	111 (20.4)	32 (13.8)	
**Alcohol status, n (%)**				
Abstainers	317 (40.9)	239 (43.9)	78 (33.6)	0.001
Moderate drinkers	364 (46.9)	250 (46.0)	114 (49.1)	
Heavy drinkers	95 (12.2)	55 (10.1)	40 (17.3)	
**Smoking status, n (%)**				
Non-smokers	441 (56.8)	318 (58.5)	123 (53.0)	0.09
Smokers	335 (43.2)	226 (41.5)	109 (47.0)	
**Frequency of night snack intake, n (%)**				
0/week	294 (37.9)	189 (34.8)	105 (45.3)	0.02
1/week	386 (49.7)	282 (51.8)	104 (44.8)	
≥2/week	96 (12.4)	73 (13.42)	23 (9.9)	
**Dietary habits, n (%)**				
Regular diet (three meals per day)	526 (67.8)	360 (66.2)	166 (71.5)	0.14
Irregular diet	250 (32.2)	184 (33.8)	66 (28.5)	
**Stress, n (%)**				
Light/moderate	459 (59.2)	322 (59.2)	137 (59.1)	0.97
Heavy	317 (40.8)	222 (40.8)	95 (40.9)	
**Work time, n (%)**				
<8 (h/day)	276 (35.6)	182 (33.4)	94 (40.5)	0.11
8–10 (h/day)	364 (46.9)	268 (49.3)	96 (41.4)	
>10 (h/day)	136 (17.5)	94 (17.3)	42 (18.1)	
**Sleep time, n (%)**				
≤8 (h/day)	559 (72.0)	380 (69.8)	179 (77.2)	0.04
>8 (h/day)	217 (28.0)	164 (30.2)	53 (22.8)	
**Hormonal parameters**				
FSH (U/L), mean ± s.d.	4.9 ± 1.9	4.9 ± 1.9	5.0 ± 1.8	0.50
LH (U/L), mean ± s.d.	3.8 ± 1.8	3.7 ± 1.7	3.9 ± 2.2	0.17
TT (ng/ml), mean ± s.d.	3.9 ± 1.4	4.1 ± 1.5	3.7 ± 1.3	<0.001
E2 (pg/ml), mean ± s.d.	41.1 ± 19.8	40.8 ± 20.8	41.8 ± 17.7	0.52
PRL (ng/ml), mean ± s.d.	10.7 ± 4.4	10.8 ± 4.6	10.4 ± 4.0	0.25
**Semen parameters**				
Abstinence time (days), mean ± s.d.	4.0 ± 1.8	4.1 ± 1.7	3.9 ± 1.9	0.15
Semen volume (ml), median (Q1, Q3)	3.6 (2.0,5.0)	3.5 (2.0,5.0)	3.6 (2.0,5.0)	0.36
Semen volume < 1.5 (ml), n (%)	50 (6.4)	34 (6.3)	16 (6.9)	0.74
Sperm concentration (× 10^6^/ml), median (Q1, Q3)	42.4 (21.3, 54.9)	40.8 (20.8, 54.2)	46.3 (24.0, 58.7)	0.03
Sperm concentration < 15 × 10^6^/ml, n (%)	117 (15.1)	87 (16.0)	30 (13.0)	0.28
Progressive motility (%), median (Q1, Q3)	22.2 (13.5, 29.1)	21.5 (12.7,28.5)	23.8 (15.8,30.3)	0.004
Progressive motility < 32%, n (%)	659 (84.9)	469 (86.2)	190 (81.9)	0.12
Normal morphology (%), median (Q1, Q3)	3.2 (2.0, 4.0)	3.1 (2.0, 4.0)	3.2 (2.5, 4.0)	0.43
Normal morphology < 4%, n (%)	549 (70.8)	385 (70.8)	164 (70.7)	0.98
Asthenoteratozoospermia, n (%)	380 (49.0)	269 (49.4)	111 (47.8)	0.97
Oligoasthenoteratozoospermia, n (%)	91 (11.7)	70 (12.9)	21 (9.1)	0.13
Leukocytic count (× 10^6^/ml), median (Q1, Q3)	0.7 (0.4, 1.0) (*n* = 313)	0.7 (0.4, 1.0) (*n* = 184)	0.7 (0.4, 1.0) (*n* = 129)	0.56
AsA (%), median (Q1, Q3)	4.6 (2.0, 5.0) (*n* = 78)	4.8 (2.0, 5.3) (*n* = 58)	3.8 (3.0, 4.0) (*n* = 20)	0.93

*BMI: body mass index. Data are presented as the mean ± s.d. if normally distributed and medians (Q1, Q3) if not for continuous variables and n (%) for categorical variables; *P* values were derived from Student’s *t*-test or the Mann–Whitney *U* test for continuous variables and from the chi-square test for categorical variables. Q1: 25th percentile. Q3: 75th percentile.*

Table 2 shows that heavy drinkers with primary infertility had higher BMI than abstainers and moderate drinkers. Smaller testis volumes were found among moderate and heavy drinkers in the secondary infertility group than among abstainers. In men with primary infertility, a statistically obvious association was detected between alcohol drinking and nighttime snack intake. Patients with heavy alcohol intake had a higher proportion of irregular diet and smoking in men with primary infertility. No significant associations between semen parameters and abstainers, moderate drinkers or heavy drinkers were seen in men with primary infertility. In the secondary infertility group, lower sperm concentrations were found in the heavy drinkers.

**TABLE 2:** Descriptive statistics according to alcohol status for the entire cohort.

**Clinical characteristics**	**Primary infertile men (*n* = 544)**				**Secondary infertile men (*n* = 232)**			
	**Abstainers (*n* = 239)**	**Moderate drinkers (*n* = 250)**	**Heavy drinkers (*n* = 55)**	***P***	**Abstainers (*n* = 78)**	**Moderate drinkers (*n* = 114)**	**Heavy drinkers (*n* = 40)**	***P***
Age (year), mean ± s.d.	29.5 ± 4.2	30.0 ± 4.6	31.0 ± 5.7	0.06	34.5 ± 5.8	35.3 ± 5.4	35.9 ± 5.1	0.35
<40, n (%)	232 (97.07)	240 (96.00)	50 (90.91)	0.11	64 (82.05)	82 (71.93)	31 (77.50)	0.26
≥40, n (%)	7 (2.93)	10 (4.00)	5 (9.09)		14 (17.95)	32 (28.07)	9 (22.50)	
BMI (kg/m^2^), mean ± s.d.	20.7 ± 3.0	20.6 ± 2.8	22.0 ± 2.6	0.003	21.1 ± 2.8	20.7 ± 2.4	21.7 ± 2.5	0.09
<24, n (%)	201 (84.10)	221 (88.40)	42 (76.36)	0.06	63 (80.77)	105 (92.11)	35 (87.50)	0.07
≥24, n (%)	38 (15.90)	29 (11.60)	13 (23.64)		15 (19.23)	9 (7.89)	5 (12.50)	
Left testis volume (cm^3^), mean ± s.d.	14.1 ± 5.1	14.0 ± 4.3	13.2 ± 2.2	0.38	16.3 ± 5.1	13.4 ± 4.5	13.1 ± 3.3	<0.001
Right testis volume (cm^3^), mean ± s.d.	15.0 ± 5.3	14.9 ± 4.2	13.3 ± 3.2	0.05	16.8 ± 6.6	14.5 ± 3.5	14.5 ± 3.5	0.002
Years of drinking, n (%)	–	7.4 ± 2.6	7.5 ± 3.1	0.75^*a*^	–	11.0 ± 4.3	11.5 ± 4.8	0.46^*a*^
**Duration of infertility, n (%)**								
1 year	128 (53.6)	135 (54.0)	28 (50.9)	0.44	62 (79.5)	80 (70.2)	32 (80.0)	0.34
2 years	67 (28.0)	64 (25.6)	11 (20.0)		9 (11.5)	15 (13.2)	2 (5.0)	
≥3 years	44 (18.4)	51 (20.4)	16 (29.1)		7 (9.0)	19 (16.6)	6 (15.0)	
**Smoking status, n (%)**								
Non-smokers	161 (67.4)	138 (55.2)	19 (34.5)	<0.001	47 (60.3)	59 (51.8)	17 (42.5)	0.17
Smokers	79 (32.6)	113 (44.8)	36 (65.5)		31 (39.7)	55 (48.2)	23 (57.5)	
**Frequency of night snack intake, n (%)**								
0/week	108 (45.2)	68 (27.2)	13 (23.7)	<0.001	40 (51.3)	49 (43.0)	16 (40.0)	0.53
1/week	109 (45.6)	150 (60.0)	23 (41.8)		29 (37.2)	54 (47.4)	21 (52.5)	
≥2/week	22 (9.2)	32 (12.8)	19 (34.5)		9 (11.5)	11 (9.6)	3 (7.5)	
**Dietary habits, n (%)**								
Regular diet (three meals per day)	171 (71.5)	160 (64.0)	29 (52.7)	<0.001	57 (73.1)	82 (71.9)	27 (67.5)	0.42
Irregular diet	68 (28.5)	90 (36.0)	26 (47.3)		21 (26.9)	32 (28.1)	13 (32.5)	
**Stress, n (%)**								
Light/moderate	145 (60.7)	146 (58.4)	31 (56.4)	0.79	50 (64.1)	68 (59.6)	19 (47.5)	0.22
Heavy	94 (39.3)	104 (41.6)	24 (43.6)		28 (35.9)	46 (40.4)	21 (52.5)	
**Work time, n (%)**								
<8 (h/day)	82 (34.31)	87 (34.80)	13 (23.63)	0.50	30 (38.46)	47 (41.23)	17 (42.50)	0.89
8–10 (h/day)	117 (48.95)	122 (48.80)	29 (52.73)		31 (39.74)	48 (42.11)	17 (42.50)	
>10 (h/day)	40 (16.74)	41 (16.40)	13 (23.63)		17 (21.80)	19 (16.67)	6 (15.00)	
**Sleep time, n (%)**								
≤8 (h/day)	167 (69.9)	177 (70.8)	36 (65.5)	0.61	58 (73.4)	90 (78.3)	31 (77.5)	0.73
>8 (h/day)	73 (30.1)	73 (29.2)	19 (34.5)		21 (26.6)	25 (21.7)	9 (22.5)	
**Hormonal parameters**								
FSH (U/L), mean ± s.d.	4.6 ± 1.9	5.0 ± 2.0	4.9 ± 2.3	0.08	5.2 ± 2.1	5.1 ± 1.3	5.2 ± 2.6	0.92
LH (U/L), mean ± s.d.	3.5 ± 1.6	3.9 ± 1.9	3.7 ± 2.4	0.06	3.7 ± 1.4	4.1 ± 3.1	3.6 ± 1.4	0.38
TT (ng/ml), mean ± s.d.	4.0 ± 1.6	4.2 ± 1.5	3.8 ± 1.8	0.15	3.5 ± 1.0	3.9 ± 1.4	3.5 ± 1.5	0.07
E2 (pg/ml), mean ± s.d.	39.3 ± 22.7	42.6 ± 20.4	38.5 ± 19.7	0.17	38.1 ± 20.0	44.5 ± 14.2	41.8 ± 21.9	0.52
PRL (ng/ml), mean ± s.d.	10.4 ± 3.5	11.1 ± 5.3	10.6 ± 5.3	0.23	11.3 ± 4.7	10.1 ± 3.6	9.6 ± 3.9	0.06
**Semen parameters**								
Abstinence time (days), mean ± s.d.	4.2 ± 1.7	4.0 ± 1.7	4.1 ± 1.9	0.79	4.1 ± 2.0	3.9 ± 1.9	3.7 ± 1.8	0.56
Semen volume (ml), median (Q1, Q3)	3.5 (2.0, 5.0)	3.5 (2.0, 5.0)	3.0 (2.1, 4.5)	0.18	3.5 (2.0,4.6)	3.5 (2.0, 5.0)	3.5 (2.6, 5.0)	0.84
Semen volume < 1.5 (ml), n (%)	13 (5.4)	15 (6.0)	6 (10.9)	0.31	5 (6.4)	8 (7.0)	3 (7.5)	0.99
Sperm concentration (× 10^6^/ml), median (Q1, Q3)	32.2 (19.4, 52.1)	35.4 (22.0, 57.2)	31.3 (17.2, 50.5)	0.46	41.9 (30.4, 70.3)	34.9 (21.1, 54.5)	36.3 (19.5, 60.7)	0.04
Sperm concentration < 15 × 10^6^/ml, n (%)	39 (16.3)	36 (14.4)	12 (21.8)	0.12	5 (6.4)	17 (14.9)	8 (18.6)	0.10
Progressive motility (%), median (Q1, Q3)	20.3 (11.7, 27.8)	21.9 (19.4, 52.1)	21.3 (10.1, 28.8)	0.46	23.4 (12.7, 31.5)	23.6 (15.3, 29.9)	25.7 (18.7, 29.3)	0.86
Progressive motility < 32%, n (%)	205 (85.8)	218 (87.2)	46 (83.6)	0.76	60 (76.9)	95 (83.3)	35 (81.4)	0.39
Normal morphology (%), median (Q1, Q3)	3.0 (2.0, 4.0)	3.0 (2.5, 4.0)	3.0 (2.0,4.0)	0.84	3.3 (2.5, 4.0)	3.0 (2.0, 4.0)	3.0 (2.0, 4.0)	0.58
Normal morphology < 4%, n (%)	169 (70.7)	175 (70.0)	41 (74.6)	0.80	53 (68.0)	81 (71.1)	30 (69.8)	0.90

*P values for derived from analysis of variance or the Kruskal–Wallis test for continuous variables and derived from the chi-square test or the Kruskal–Wallis test for categorical variables, if not otherwise indicated. ^*a*^Student’s *t*-test. Q1: 25th percentile. Q3: 75th percentile.*

As shown in Table 3, no statistically significant association was found between semen parameters in the crude models for men with primary infertility or men with secondary infertility. After adjustment for age, BMI, abstinence time, smoking, duration of infertility, nighttime snack intake, dietary habits and sleep time, heavy drinking was observed to be negatively correlated with sperm concentration in the secondary infertility group, while no relationship was detected between drinking and other semen parameters (volume, total sperm count, sperm motility, sperm morphology). Meanwhile, we also reanalyzed data that alcohol intake as a continuous variable and found that sperm concentration (*r* = −0.15, *P* = 0.02) were negatively correlated to alcohol consumption in secondary infertile men, while no significant correlation to semen parameters in primary infertile men (Supplementary Table S1).

**TABLE 3:** Odds ratios (95% confidence intervals) for abnormal semen parameters across different levels of alcohol consumption.

**Clinical characteristics**	**Primary infertile men (*n* = 544)**			**Secondary infertile men (*n* = 232)**		
	**Abstainers (*n* = 239)**	**Moderate drinkers (*n* = 250)**	**Heavy drinkers (*n* = 55)**	**Abstainers (*n* = 78)**	**Moderate drinkers (*n* = 114)**	**Heavy drinkers (*n* = 40)**
**Semen volume**						
Crude *P*	ref	1.11 (0.52; 2.38) 0.79	2.13 (0.77; 5.88) 0.15	ref	1.25 (0.40; 3.89) 0.70	1.18 (0.27; 5.23) 0.82
Adjusted *P*	ref	0.90 (0.41; 2.00) 0.80	1.35 (0.43; 4.20) 0.61	ref	1.26 (0.39; 4.10) 0.70	1.20 (0.25; 5.71) 0.82
**Sperm concentration**						
Crude *P*	ref	0.86 (0.53; 1.41) 0.56	1.43 (0.69; 2.96) 0.33	ref	2.56 (0.90; 7.26) 0.08	3.10 (0.92; 10.48) 0.07
Adjusted *P*	ref	0.87 (0.52; 1.45) 0.58	1.44 (0.65; 3.16) 0.37	ref	2.93 (0.99; 8.70) 0.05	3.72 (1.04; 13.37) 0.04
**Total count**						
Crude *P*	ref	0.82 (0.49; 1.39) 0.47	1.51 (0.71; 3.20) 0.29	ref	1.78 (0.70; 4.51) 0.23	1.79 (0.56; 5.74) 0.33
Adjusted *P*	ref	0.83 (0.49; 1.43) 0.51	1.47 (0.68; 3.45) 0.30	ref	2.09 (0.78; 5.61) 0.14	2.04 (0.59; 6.99) 0.26
**Progressive motility**						
Crude *P*	ref	1.13 (0.67; 1.90) 0.65	0.85 (0.38; 1.89) 0.69	ref	1.50 (0.73; 3.09) 0.27	1.70 (0.62; 4.70) 0.31
Adjusted *P*	ref	1.15 (0.68; 1.96) 0.61	0.82 (0.35; 1.92) 0.64	ref	1.44 (0.69; 3.01) 0.33	1.73 (0.61; 4.91) 0.31
**Total motility**						
Crude *P*	ref	0.87 (0.59; 1.29) 0.49	0.91 (0.48; 1.74) 0.78	ref	1.04 (0.57; 1.89) 0.91	1.04 (0.47; 2.31) 0.92
Adjusted *P*	ref	0.89 (0.59; 1.33) 0.55	0.95 (0.48; 1.90) 0.89	ref	0.91 (0.49; 1.70) 0.77	0.93 (0.41; 2.14) 0.87
**Normal morphology**						
Crude *P*	ref	1.01 (0.68; 1.50) 0.97	1.03 (0.52; 2.06) 0.93	ref	1.17 (0.61; 2.23) 0.64	0.98 (0.42; 2.28) 0.96
Adjusted *P*	ref	1.10 (0.73; 1.67) 0.64	1.27 (0.61; 2.64) 0.52	ref	1.08 (0.55; 2.14) 0.82	1.09 (0.45; 2.68) 0.85

*The reference group for the analyzed parameters was men with normal semen quality values according to the WHO: semen volume ≥ 1.5 ml, sperm concentration ≥ 15 **×** 10^6^/ml, progressive motility sperm ≥ 32 **×** 10^6^/ml, motility morphology ≥ 4%. Crude: unadjusted model; Adjusted: model adjusted for age, BMI, duration of infertility, abstinence time, smoking, night snack intake, dietary habits, and sleep time.*

## Discussion

Poor semen quality (low sperm concentration and total sperm count, abnormal sperm motility and morphology) is known to be a key factor in male fertility. The aim of this study was to investigate the impact of alcohol intake on semen parameters among infertile men. Our findings suggest that heavy drinking was positively correlated with low sperm concentrations in men with secondary infertility. However, both crude and adjusted model analyses showed that alcohol intake in primary infertile men was not associated with semen parameters.

So far, no studies have reported detailed alcohol consumption among primary and secondary infertile men from China. In the present study, we demonstrated that the consumption of ≥9 units/week of alcohol intake (heavy drinking) in men with secondary infertility was associated with lower sperm concentrations than in the men who consumed <9 units/week of alcohol (moderate drinkers) or those who consumed no alcohol (abstainers). We also identified a longer duration of alcohol consumption in drinkers with secondary infertility compared with the men with primary infertility. Interestingly, a similar phenomenon regarding decreased sperm concentration was observed in 20 men with alcohol dependence syndrome (Kucheria et al., 1985). Moreover, a 6 year follow-up study showed a progressive impact of heavy chronic alcoholic intake on sperm concentration (from 89 × 10^6^/mL to 10 spermatozoa) (Sermondade et al., 2010). Therefore, alcohol intake may more commonly contributes to secondary infertility than to primary infertility, since the impact of alcohol consumption increases over time. However, our findings differ from the study by Martini et al., who declared that alcohol intake did not seem to be associated with abnormal semen parameters (Martini et al., 2004). In previous studies showing that alcohol consumption does not play any role in semen quality or male fertility, the majority of the reports do not separate men with secondary infertility from men with primary infertility. In fact, the related clinical characteristics associated with alcohol consumption in men with secondary infertility cannot be disregarded.

The mechanisms involved in the association between alcohol intake and reproductive ability were investigated using *in vitro* and *in vivo* models. Alcohol consumption was positively correlated with sperm apoptosis and spermatogenesis defects that induced low total sperm counts and sperm concentrations (Jana et al., 2010; Sansone et al., 2018). In the study of Pajarinen and Karhunen (1994) more than one-third of the heavy drinking (consuming >5 units of alcohol per week) men had partial or complete spermatogenic arrest. Moreover, dramatically decreased testicular weight was observed in heavy-drinkers compared with abstainers. Notably, testosterone synthesis was blocked by alcohol injection in adult male mice by decreasing key gene expression (StAR, 3β-HSD, and 17b-HSD) in the androgen synthesis pathway (Jana et al., 2010). Thus, the abnormal serum hormone levels after alcohol consumption may lead to progressive testicular damage. However, in this study, no difference between hormonal serum levels in non-drinkers and drinkers may be explained by a direct effect on the testes instead of the hypothalamic-pituitary-testicular axis, which is consistent with the observed findings by Sermondade et al. (2010).

Studies have evaluated the correlation between semen quality and age, BMI, duration of infertility, dietary habits, stress, work time, and sleep time; all of these variables were used in this study as confounders. With the age at marriage increasing, semen parameters have changed around the world over the last decade (Zegers-Hochschild et al., 2009). Thus, researchers have focused on the effects of aging on male fertility. Compared with semen quality for men younger than 40 years of age, semen quality is significantly decreased in men aged 40 years and older (Katib et al., 2014; Ramasamy et al., 2015). Older age (more than 40 years) was associated with an increased rate of abnormality in semen volume, sperm motility, sperm vitality, and sperm kinematics (Veron et al., 2018). The semen parameters of the men in couples trying to achieve a pregnancy might have changed due to the increased age at marriage. In this study, as expected, men with secondary infertility were older than men with primary infertility, consistent with other reports (Walsh et al., 2009). However, there were no differences in patient age between abstainers and drinkers. Previous research has reported that increasing BMI correlates with decreasing semen parameters (Bieniek et al., 2016). A large meta-analysis showed that obese men had an increased percentage of abnormal sperm morphology (Campbell et al., 2015). Our study suggests that there were no obvious differences in BMI between the primary and secondary infertility groups, which is consistent with the results reported by Sahin et al. (2017). Notably, only in the primary infertile group did we observe that BMI was markedly higher in patients with heavy alcohol consumption than in abstainers and moderate drinkers, although there was no change in semen parameters among patients with different drinking patterns. One possible explanation for this finding is that abnormal dietary habits (high nighttime snack consumption and an irregular daily diet) may play a key role in body weight (Tanner et al., 2019). A number of studies have investigated whether alcohol intake could change testicular development, especially testicular volume, and they have reported decreased testicular volume in rats with long-term alcohol consumption (Calleja Escudero et al., 1997). Consistent with the above report, testicular weights were reduced by 37% after chronic ethanol treatment in mice (Anderson et al., 1989). In the present study, both left and right testicular volume in secondary infertile men with heavy drinking were reduced when compared with the volume in non-drinkers, which may indicate a risk of low sperm concentration in drinkers. Similarly, a comparison of the duration (years) of infertility between men with primary and secondary infertility has been reported by several studies. However, only the mean value was provided in these studies. In this study, the duration (years) was divided into 1 year, 2 years, and 3 or more than 3 years; a decreased duration of infertility (years) was found in men with secondary infertility compared with men with primary infertility, which may be due to less time being spent preparing for pregnancy in China than in other countries.

The limitations of our study are that, first, the study was cross-sectional and did not provide a possible direction for the observed relationships. Second, although data on alcohol consumption were obtained from the questionnaire, exposure misclassification and recall bias may have occurred in this study. Third, the general fertile male population was not included. Fourth, sex hormones were not performed with mass spectrometry. Moreover, the number of participants, especially in the case of heavy drinkers, was rather small, and all of them were recruited from one fertility clinic, increasing selection bias that might have affected the results. Finally, due to the small number of men with primary or secondary infertility reporting an intake of ≥9 units/week, the potential effect of high alcohol intake should be interpreted with caution.

The major strength of our study was that we were able to investigate the differences in alcohol consumption between men with primary and secondary infertility. Meanwhile, we also were able to evaluate potential confounders, including age, BMI, diet pattern, sleep duration and stress, which may influence drinking behaviors and semen quality.

In summary, we present evidence that heavy alcohol consumption in men with secondary infertility, but not primary infertility is correlated with lower sperm concentrations. This study fills an important research gap by revealing the link between alcohol consumption in infertile men, including patients with primary and secondary infertility, and semen quality in China.

## Data Availability Statement

All datasets presented in this study are included in the article/Supplementary Material.

## Ethics Statement

The studies involving human participants were reviewed and approved by The First Affiliated Hospital of USTC Ethical Committee, Anhui, China. The patients/participants provided their written informed consent to participate in this study.

## Author Contributions

SB designed the research study and wrote the manuscript. TG, YW, and SB contributed to the data acquisition. TG, XT, YW, LZ, WL, XX, YZ, YZZ, XH, and BX analyzed the data. All authors contributed to the article and approved the submitted version.

## Conflict of Interest

The authors declare that the research was conducted in the absence of any commercial or financial relationships that could be construed as a potential conflict of interest.

## Supplementary Material

TABLE S1 Intragroup correlation for semen parameters and alcohol intake.
