# Supplementary material for: Association of Alcohol Intake and Semen Parameters in Men With Primary and Secondary Infertility: A Cross-Sectional Study
Source: Front Physiol. 2020 Sep 11;11:566625. doi: 10.3389/fphys.2020.566625 (PMC7517893; doi:10.3389/fphys.2020.566625)
Supplement: TABLE S1 — Intragroup correlation for semen parameters and alcohol intake. [file Table_1.DOCX]

**Supplementary Table S1** Intragroup correlation for semen parameters and alcohol intake.

| Clinical characteristics | Primary infertile men (n=544) | | Secondary infertile men (n=232) | |
| --- | --- | --- | --- | --- |
|  | r | *P* | r | *P* |
| Semen volume | -0.04 | 0.33 | 0.03 | 0.62 |
| Sperm concentration | 0.01 | 0.79 | -0.15 | 0.02 |
| Total count | -0.02 | 0.57 | -0.09 | 0.17 |
| Progressive motility | 0.05 | 0.28 | 0.01 | 0.94 |
| Total motility | 0.04 | 0.42 | 0.01 | 0.94 |
| Normal morphology | 0.05 | 0.30 | -0.02 | 0.82 |

r = spearman’s correlation coefficient
